# Three-dimensional finite element analysis of full-arch implant-supported dental restorations: complete vs. segmented frameworks

**DOI:** 10.3389/fbioe.2026.1759007

**Published:** 2026-04-30

**Authors:** Eduardo Anitua, Mikel Armentia, Luis Saracho

**Affiliations:** 1 Eduardo Anitua Foundation, Vitoria, Spain; 2 R&D Department, Biotechnology Institute I mas D S.L., Vitoria, Spain

**Keywords:** dental implants, finite element analysis (FEA), framework segmentation, full-arch restoration, peri-implant bone stress

## Abstract

**Purpose:**

Given the reported clinical advantages of segmented implant-supported mandibular frameworks, multiple segmentation patterns were evaluated and compared with a complete (one-piece) framework to determine their effects on peri-implant bone stress.

**Methods:**

Thirteen cases were studied via 3-D finite element analysis (FEA), combining complete and segmented full-arch frameworks across different implant layouts. Screws were preloaded according to manufacturer recommendations. Cortical and trabecular bone were modeled as homogeneous, isotropic, linearly elastic materials, and a fully osseointegrated implant–bone interface (shared-node mesh) was assumed. A static vertical load of 200 N was applied in three separate load cases: first molar, first premolar, and bilateral incisors. The primary outcome was peak von Mises equivalent stress in the peri-implant bone surrounding each implant as a comparative indicator of mechanical demand.

**Results:**

When the load fell between two splinted implants (e.g., lateral segment without cantilever), peri-implant bone stresses were similar for complete and segmented frameworks. Under premolar loading, the complete framework yielded the lowest stresses; segmenting with a mesial cantilever on the lateral segment approximately doubled stresses at the adjacent implant, while placing a distal cantilever on the central segment increased stresses near the anterior support by up to ∼7× versus the complete configuration. Eliminating cantilevers by moving support from 5 to 4 closely approached the complete framework. Incisor loading challenged the central span: supports at 3–3 produced markedly higher stresses than complete frameworks; shifting supports to 2–2 reduced stresses but did not match the complete design, whereas triangulating the central span (3–2–3) further lowered stresses and approached complete-framework levels.

**Conclusion:**

Segmented frameworks are biomechanically viable provided segmentation avoids central-span cantilevers, preferentially places any unavoidable cantilever on a straight lateral segment, and—when feasible—adds an anterior support to triangulate the central span or eliminates cantilevers by advancing support distally. With a properly planned segmented framework, peri-implant bone stresses can remain close to those of complete frameworks under the loading conditions investigated.

## 1 Introduction

Rehabilitation of the edentulous mandible with an implant-supported prosthesis is widely performed and is associated with high survival rates (Schimmel et al., 2023; Srinivasan et al., 2023). Implants can be placed in a range of configurations, each with specific advantages and limitations that have been extensively discussed (Maló et al., 2003; Nedir et al., 2004; Anitua et al., 2008; Bedrossian and Bedrossian, 2019; Carossa et al., 2022; Menini et al., 2022). However, this discussion has often diverted attention from the prosthetic superstructure itself and its impact on restoration biomechanics—particularly the response of the peri-implant bone.

With respect to the prosthesis, clinicians have traditionally opted for a complete (one-piece) framework, as the resulting superstructure is inherently stiffer and more robust. Such increased rigidity has been associated with reduced peri-implant bone stresses (Dayan and Geckili, 2021; Kelkar et al., 2021; Grande et al., 2022; Tribst et al., 2022). Nevertheless, designing a sectioned (segmented) framework has several clinical advantages that make it an increasingly attractive option (Marin et al., 2015; Zaki and Bahig, 2023).

On the one hand, while splinting all units in a single piece can be expected to increase global stiffness and thereby reduce peri-implant bone stresses, excessive rigidity is not invariably beneficial. An overly rigid framework may dampen physiological mandibular flexure, which, rather than being favorable, could diminish functional stimulation of the mandible and concentrate stresses at specific sites, potentially promoting marginal bone loss (Law et al., 2012; Mijiritsky et al., 2022).

On the other hand, a frequent practical limitation of complete frameworks is misfit at one or more implant connections, arising from inaccuracies in capturing implant positions (e.g., scanning/impression errors) and/or manufacturing tolerances (Nagata et al., 2021; Anitua et al., 2024; Asavanant et al., 2025). Such misfit may compromise the seal of the Implant–Abutment Connection (IAC) through the formation of microgaps, creating conditions conducive to bacterial ingress and proliferation within the IAC, with potential progression to peri-implantitis, bone loss, and implant failure (Lindhe et al., 1992; Hermann et al., 1997; 2000; Oh et al., 2002; Broggini et al., 2003; Steinebrunner et al., 2005; Weng et al., 2008; Do Nascimento et al., 2009; Boynueğri et al., 2012; Rismanchian et al., 2012; Carinci et al., 2016). In addition, tightening a misfitting complete framework can impose undesirable tensile loads on the implant and its surrounding bone. Finally, if a residual misfit or gap persists after screw tightening, progressive loss of screw preload may occur under cyclic masticatory loading, ultimately leading to prosthetic screw loosening (Rismanchian et al., 2012; Benjaboonyazit et al., 2019; Naseri et al., 2021). Screw loosening, in turn, degrades the mechanical performance of the restoration and can disrupt the connection seal—facilitating microleakage and bacterial contamination—or even culminate in screw fracture (Secatto et al., 2017; Siadat et al., 2018; Singh et al., 2022).

Despite the growing clinical adoption of segmented full-arch frameworks, the biomechanical evidence base still provides limited, pattern-specific guidance on which segmentation schemes are mechanically acceptable. Most prior work has either treated segmentation as a binary choice (one-piece vs. segmented) or focused on other variables (e.g., framework materials, implant number/angulation, cantilever length) without systematically isolating the location of framework splits, the segment that bears any unavoidable cantilever, and the role of central-span support strategies. As a result, clinicians lack biomechanics-driven criteria to judge whether a planned segmentation pattern is likely to preserve peri-implant mechanical conditions comparable to a one-piece framework under the investigated loading scenarios.

Therefore, the objective of this *in silico* three-dimensional finite element study was to quantify how specific segmentation patterns affect peri-implant bone stress, compared with a conventional one-piece framework, across clinically realistic implant layouts and representative loading sites. While clinical issues such as prosthetic misfit, microgap formation/bacterial leakage, and cyclic preload loss motivate interest in segmentation, these phenomena were not explicitly modeled here (no imposed misfit or leakage modeling; no cyclic loosening simulation). Accordingly, the study addresses a narrower biomechanical question: how the segmentation pattern (split locations, cantilever-bearing span, and central-span support strategy) influences peri-implant bone stress under the investigated loading conditions. Unlike prior finite element analysis (FEA) studies, this work provides a configuration-matched mapping of multiple segmentation patterns under different implant layouts and loading sites, thereby isolating the mechanical role of split location, cantilever-bearing span, and central-span support strategy.

It was hypothesized that: (H1) when the occlusal load is applied between two splinted supports within a straight span (i.e., without a cantilever on the loaded span), segmented frameworks would yield peri-implant bone stresses comparable to those of one-piece frameworks; (H2) when segmentation results in a loaded cantilever, peri-implant bone stresses would be higher than those of one-piece frameworks; and (H3) under anterior loading of the central span, triangulating the central segment by adding an anterior support (e.g., 3–2–3) would reduce stresses compared with non-triangulated central support schemes and towards one-piece levels.

## 2 Materials and methods

**FIGURE 1:**
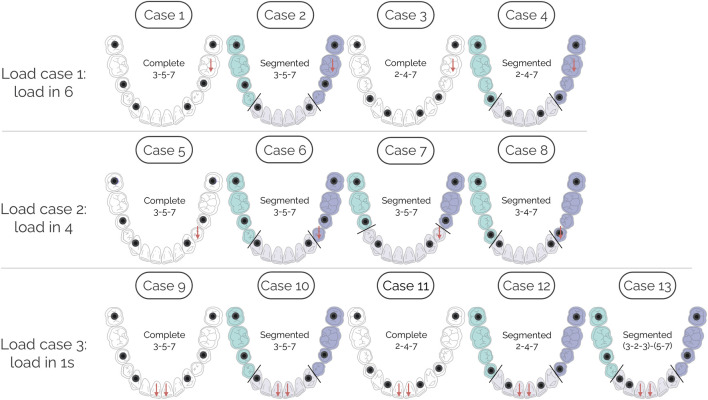
Implant layouts and framework segmentations in an edentulous mandible for each of the three load cases analyzed. Load case 1 (vertical load at 6): cases 1–4. Load case 2 (vertical load at 4): cases 5–8. Load case 3 (vertical load at the two 1s): cases 9–13.

Different load cases, implant layouts, and framework segmentations were analyzed in an edentulous mandible. As shown in Figure 1, three load cases were studied. In the first load case, four frameworks were evaluated under a vertical load applied to the first molar (tooth positions 36 and 46, according to the FDI two-digit system, ISO 3950; hereafter, only the second digit is reported, as both mandibular quadrants are considered). Case 1 consists of a complete framework over three implants placed at 3, 5, and 7. Case 2 uses the same implant arrangement, but the framework is sectioned flush with the implant at 3, leaving a central segment supported at 3 and two lateral segments supported at 5 and 7, with a mesial cantilever at 5. Case 3 consists of a complete framework supported by implants at 2, 4, and 7. Case 4 used the same implant arrangement, but the framework is sectioned flush with the implant at 4, creating a distal cantilever borne by the central segment supported at 2.

In the second load case, the effect of a vertical load on position 4 was evaluated by Cases 5–8. Case 5 has the same arrangement as Case 1, i.e., a complete prosthesis supported on implants at 3, 5, and 7. Case 6 corresponds to the same arrangement as Case 2, i.e., a central segment from 3 to 3 and lateral segments from 5 to 7, with a mesial cantilever in 5. Case 7 is also supported by implants in 3, 5, and 7 but now segmenting the framework flush with the implant in 5, with a distal cantilever in 3. In Case 8 cantilevers are completely eliminated by supporting the framework on implants in 3, 4, and 7 and sectioning the prosthesis between 3 and 4.

Finally, in the third load case, the central segment was analyzed by applying a vertical load equally distributed between the two 1s. In Case 9, a complete prosthesis is supported on implants at 3, 5, and 7, as in Case 1. In Case 10, as in Case 2, a central segment is supported on implants placed in both 3s (without cantilevers), while lateral segments are supported on implants in 5 and 7, leaving a mesial cantilever at 5. In Case 11, the same case of complete prosthesis on implants 2, 4, and 7 presented in Case 3 is used. In Case 12, the same implant arrangement is used as in Case 11 but, in this case, the prosthesis is sectioned flush with 4, with the cantilever being supported by the central segment in 2 distally. In Case 13, the arrangement of Case 10 is used, i.e., lateral segments from 5 to 7 with mesial cantilevers supported by implant in 5 and a central segment from 3 to 3 (without cantilevers), but this time adding an extra implant in 2.

For the subsequent FEA, CAD geometry of human mandible was modelled by using NX 8.5 software. The mandible model consisted of a trabecular bone fill and 1.5 mm cortical bone shell. In this mandible model, several BTI INTERNA 3.0 implants of 7.5 mm length were placed. 3 mm diameter implants (IIP3CA3075) were used for implants placed in positions 1 to 5, while 3.3 mm diameter implants (IIP3CA3375) were used for implants placed in position 7. On these implants, transepithelial abutments of 3 mm height and 4.1 mm prosthetic platform (INTMIP330) were mounted. Finally, the prosthesis was mounted on top of these transepithelial abutments, screwed to the abutments by means of prosthetic screws (TTMIR).

To standardize peri-implant evaluation and mesh refinement across all configurations, two concentric peri-implant bone shells were generated around each implant within the surrounding bone: (i) an inner region of interest (ROI) consisting of a 0.5-mm-thick radial peri-implant bone layer used for stress/strain extraction, and (ii) an additional 0.5-mm-thick concentric transition layer created to facilitate a smooth mesh-size gradation from the refined peri-implant region to the coarser bone mesh away from the implants.

**FIGURE 2:**
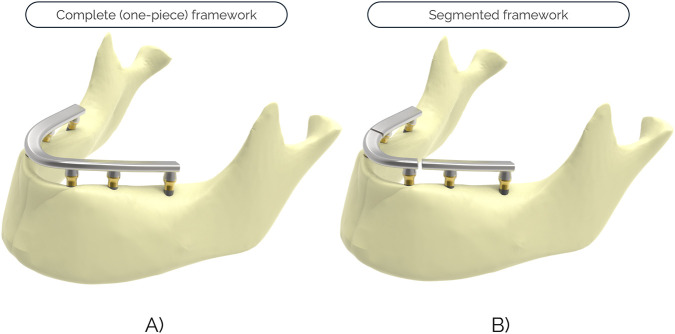
Examples of the CAD geometry of the prosthetic frameworks: **(A)** Complete (one-piece) framework corresponding to Case 1. **(B)** Segmented framework corresponding to Case 2.

Figure 2 shows Cases 1 and 2 as examples of the CAD geometry for a complete (one-piece) framework and a segmented one, respectively. Before the FEA calculations, certain simplifications were introduced in the model. On the one hand, the external threads of the implants were not modeled in order to avoid the influence of its rotational position (with respect to the implant axis) on the stresses in the peri-implant bone. On the other hand, all the threads of the screws (and the internal threads of the implant) were modeled as cylindrical, simplifying the model and avoiding possible errors in the calculations, leading to minimal differences in terms of stresses with respect to their real shape (Armentia et al., 2020). Finally, the mandibular bone was sectioned in two locations, leaving the condyles outside the model.

Regarding FEA, the model was imported into ANSYS Workbench® 19R1 software for the analysis, including pre- and post-processing. The implants, transepithelial abutment bodies, and prosthetic frameworks were made of commercially pure grade 4 titanium (CP4). These components were modeled as homogeneous, isotropic, linearly elastic materials with an elastic modulus of E=103 GPa and a Poisson’s ratio of ν=0.35 . The screws were made of Ti-6Al-4V ELI titanium alloy (grade 5) and were also modeled as homogeneous, isotropic, linearly elastic, using the same elastic modulus and a Poisson’s ratio of ν=0.31 . The chemical composition of the metallic materials is summarized in Table 1.

**TABLE 1:** Chemical composition of materials used in implants and prosthetic components according to (“Standard Specification for Unalloyed Titanium, for Surgical Implant Applications” (ASTM F67-13, 2017); “Standard Specification for Wrought Titanium-6Aluminum-4Vanadium ELI (Extra Low Interstitial) Alloy for Surgical Implant Applications” (ASTM F136, 2021)).

Ti 6Al 4 V ELI (TI GR5)		TI CP4	
Composition	Wt. %	Composition	Wt. %
Al	5.5–6.5	N (max)	0.05
V	3.5–4.5	C (max)	0.08
Fe (max)	0.25	Fe (max)	0.5
O (max)	0.13	O (max)	0.4
C (max)	0.08	H (max)	0.0125
N (max)	0.05	-	-
H (max)	0.012	-	-

Cortical and trabecular bone were modeled as homogeneous, isotropic, linearly elastic materials, with E=13,700 MPa and ν=0.28 for cortical bone and E=1,370 MPa and ν=0.30 for trabecular bone. This first-order approximation is commonly adopted in implant dentistry FEA for comparative/parametric investigations (Falcinelli et al., 2023). Bone is inherently heterogeneous and anisotropic, and more detailed material descriptions (e.g., orthotropic bone properties) may influence the absolute magnitude and local peaks of peri-implant stresses (Afazal et al., 2025). In the present study, the aim was to isolate the mechanical effect of framework segmentation pattern and support strategy under consistent assumptions across all cases; therefore, results are interpreted primarily as relative comparisons between configurations rather than as patient-specific absolute stress predictions.

Contacts were defined as frictional, with a coefficient of friction of 0.17 for screw–implant and screw–transepithelial abutment interfaces, and 0.21 for implant–transepithelial abutment and transepithelial abutment–framework interfaces, as determined in a previous work (Armentia et al., 2020).

Zero displacement conditions were applied on the aforementioned cuts performed in the mandible in order to avoid rigid body motion even though the stiffness of the model may be increased. The model was meshed by means of second order tetrahedra of maximum size 0.25 mm in the peri-implant bone, implants and screws, 0.35 mm in the transepithelial abutments, 0.5 mm in the prosthesis, and 1 mm in the bone away from the implants. To favor the transition of elements in the bone, transition layers were meshed with elements of a maximum size of 0.4 mm. The implant–bone interface was assumed fully osseointegrated and was modeled as a perfectly bonded interface using a conformal shared-node mesh (shared topology), preventing any relative motion or separation at the interface.

**FIGURE 3:**
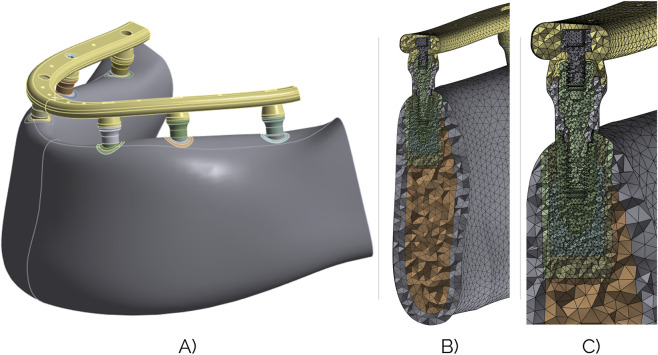
**(A)** FE model with the aforementioned simplifications. **(B)** Section view of the meshed model. **(C)** Close-up view of the meshed implant assembly.

The analyses consisted of three steps. In the first step, preloads were applied to the transepithelial abutments using the Bolt Pretension tool, simulating the 35Ncm tightening torque recommended by the manufacturer. Specifically, 688 N were applied, calculated using the Motosh formula (Motosh, 1976; Bickford, 2008; Armentia et al., 2020). In the second step, the preloads were applied on the prosthetic screw that joins the prosthesis along with each one of the transepithelial abutments by using the same tool mentioned in the previous step. In this case, according to the same formula, the manufacturer’s recommended torque, i.e. 20Ncm, corresponds to 566 N. As it is previously mentioned, three load cases were analyzed, with all of them being vertical loads of 200 N (Tribst et al., 2017; 2022; Kumari et al., 2020; Masoomi and Mahboub, 2024) and shifting only the load application point. Accordingly, in the third and last step, this load was applied. In Cases 1–4 the load was applied in position 6, in Cases 5–8 the load was applied in position 4 and in Cases 9–13 the load was applied equally distributed between the two 1s (left and right). Figure 3 shows the FE model and a detailed section view of the mesh.

The primary outcome was the peak von Mises equivalent stress in the peri-implant bone, extracted as the maximum value within the predefined 0.5-mm peri-implant ROI surrounding each implant. As complementary descriptors of the peri-implant mechanical state, within the same ROI the maximum and minimum principal stresses (σ_1_ and σ_3_) and elastic strain measures were extracted, including equivalent elastic strain and principal elastic strains (ε_1_ and ε_3_). Because these metrics describe different aspects of the stress/strain state, their peak values are not required to occur at the same spatial location within the ROI; therefore, the maximum (and minimum, where applicable) for each metric was obtained independently within the ROI.

## 3 Results

**FIGURE 4:**
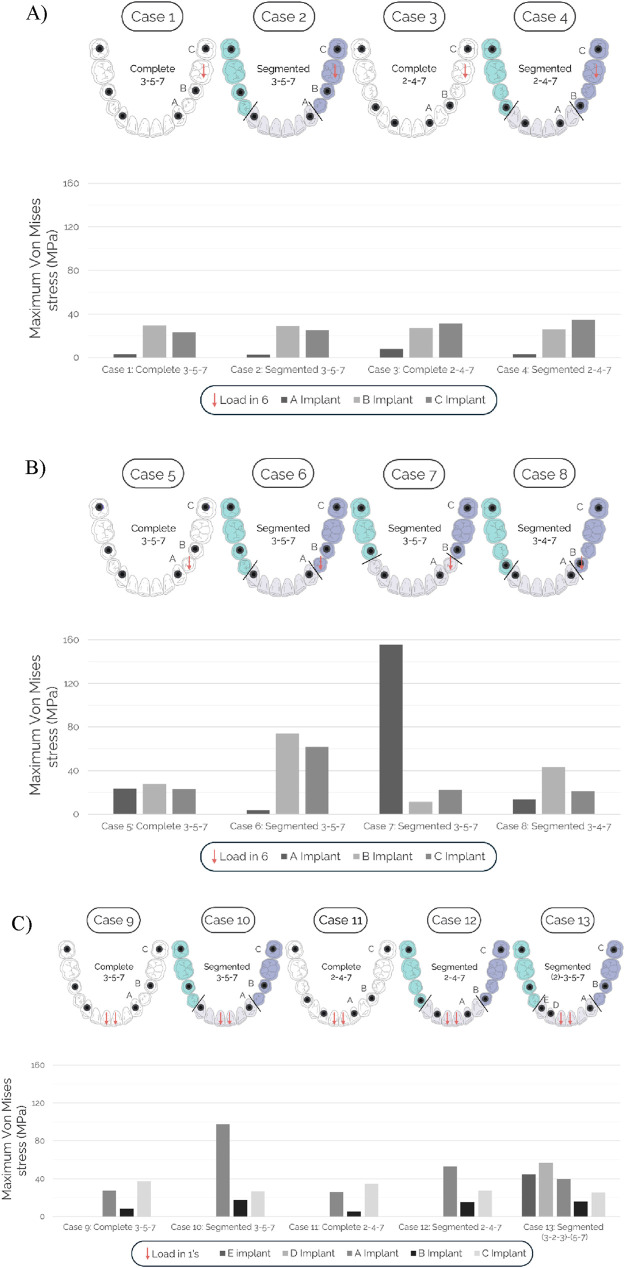
Maximum von Mises stresses in the peri-implant bone surrounding each implant for each of the three load cases analyzed. **(A)** Load case 1 (vertical load at 6): cases 1–4. **(B)** Load case 2 (vertical load at 6): cases 5–8. **(C)** Load case 3 (vertical load at the two 1s): cases 9–13.

Peak peri-implant bone stresses are summarized in Figure 4, with von Mises equivalent stress used as the primary comparative metric. In addition to von Mises stress, maximum and minimum principal stress components (σ_1_ and σ_3_) and elastic strain measures (ε_eq_, ε_1_ and ε_3_) were extracted within the same peri-implant ROI for each implant (see Materials and Methods) and are provided in Supplementary Table S1. To maintain clarity in the main text and figures, the results section focuses on von Mises stress, while the supplementary metrics are used to corroborate the configuration-driven trends.

In Figure 4A, all cases loaded at position 6 (Cases 1–4) exhibited similar peri-implant bone stress levels. Among Cases 5–8 (load at position 4, see Figure 4B), Case 5 showed the lowest maxima across all implants. In Case 6—with a mesial cantilever at 5 on the lateral segment—peak peri-implant von Mises stress around implant 5 increased from 28 to 74 MPa (≈2.6×) relative to Case 5. By contrast, in Case 7, which features a distal cantilever at 3 on the central segment, stresses around implant 3 increased by nearly seven-fold versus Case 5 (from 23 to 156 MPa). Case 8, with no cantilevers, most closely approached Case 5, showing a slight rise around implant 4 and slight reductions elsewhere.

For incisor loading (Cases 9–13, see Figure 4C), Cases 9 and 11 (complete frameworks) yielded the lowest stress levels. Case 10 (central segment supported at 3–3) showed a marked increase. Case 12 (central segment supported at 2–2) reduced stresses substantially relative to Case 10, though it did not match the complete frameworks. Case 13 (central span triangulated at 3–2–3) further lowered stresses and approached, but did not reach, the minima observed with the complete configurations.

## 4 Discussion

Figure 4A shows that a load applied at position 6 that is not borne by a cantilever does not compromise the system, irrespective of whether the framework is complete or sectioned and whether support is provided at 4–7 or 5–7. In other words, when the load falls between two splinted implants, peri-implant bone stresses remain moderate regardless of segmentation. This observation aligns with previous reports demonstrating the opposite condition—namely, that the presence and extension of a cantilever substantially increase stresses on the supporting implants and peri-implant bone (Maminskas et al., 2016; Suedam et al., 2016; Batista et al., 2017; Paes-Junior et al., 2019; Kumari et al., 2020; Anitua et al., 2022).

From Figure 4B, when the framework is sectioned such that the cantilever is borne by the central segment, stresses increase markedly relative to the complete framework. By contrast, when the cantilever lies on the lateral (straight) segment, the stress increase is more moderate. Eliminating the cantilever altogether by advancing the implant from 5 to 4 (cf. Case 8) yields stress levels that most closely resemble those of the complete configuration (cf. Case 5). Accordingly, if a sectioned framework is employed, cantilevers should be avoided whenever possible. Moreover, central-segment cantilevers appear more detrimental than lateral-segment cantilevers, in agreement with previous finite element studies showing that distal or centrally borne cantilevers lead to the highest peri-implant stress concentrations, whereas mesial or laterally borne cantilevers distribute load more favorably (İleri et al., 2025).

Clinically, this effect may be further accentuated by the frequent use of implants of larger diameter in posterior positions, which improves stress distribution and reduces cortical bone strain (Silva-Neto et al., 2014; Muangsisied et al., 2021). Therefore, when segmentation is required, placing any unavoidable cantilever on a lateral segment supported by a wider-diameter implant may provide a biomechanically safer configuration.

Figure 4C indicates that incisor loading markedly challenges the central segment, which—owing to its curvature and distance to the supports—behaves effectively as a cantilever. If a sectioned framework is selected, supporting the central segment on implants at 2–2 rather than 3–3 can reduce this effective cantilever. However, the 2–2 configuration may introduce a loaded distal cantilever at 3 or 4 borne by the central span, which is unfavorable for peri-implant bone stresses, as previously noted. Consequently, when retaining supports at 3–3, stresses can be mitigated by adding an auxiliary support and triangulating the central segment (3–2–3).

Taken together, these observations allow a direct assessment of the proposed hypotheses. Overall, the results were consistent with the study hypotheses under the investigated loading conditions. H1 was supported, as configurations in which the occlusal load was applied between splinted supports without a cantilever on the loaded span (e.g., molar loading in Figure 4A) showed peri-implant stress levels that were similar for segmented and one-piece frameworks. H2 was supported, since cases in which segmentation produced a loaded cantilever (Figure 4B) exhibited higher peri-implant stresses than the corresponding one-piece configuration, with the most pronounced increases occurring when the cantilever was borne by the curved central span. H3 was supported, as anterior loading of the central span showed that adding an anterior support to triangulate the segment (3–2–3; Case 13 in Figure 4C) reduced peri-implant stress demand compared with non-triangulated central support schemes (e.g., 3–3 in Case 10) and moved the response towards the one-piece configurations, although one-piece frameworks still yielded the lowest stresses in absolute terms.

Notably, these configuration-driven trends were obtained under idealized modeling assumptions. Cortical and trabecular bone were represented as homogeneous, isotropic, linearly elastic materials; because bone is inherently heterogeneous and anisotropic, more detailed descriptions (e.g., orthotropic or CT-based heterogeneous properties) may influence the absolute magnitude and local peaks of peri-implant stresses. In addition, the present findings are strictly valid for the investigated static axial (vertical) loading condition of 200 N; oblique and/or cyclic loading may modify stress magnitudes and distributions and should be addressed in future work.

The implant–bone interface was assumed to be perfectly osseointegrated (fully bonded/shared-node topology). Zero-displacement constraints were applied at the mandibular cut surfaces (condyles excluded) to prevent rigid-body motion; this may increase global stiffness and affect absolute peak values, although the constrained regions were remote from the peri-implant ROI and were applied identically across cases. Finally, only one implant–abutment connection/component set, framework/implant material set, and specific contact/friction and preload definitions were considered; therefore, absolute stress/strain magnitudes should be interpreted with caution and extrapolation to other systems, materials, and geometries should be made carefully. Under these consistent assumptions, the primary interpretation is the comparative ranking across configurations rather than patient-specific absolute predictions.

**FIGURE 5:**
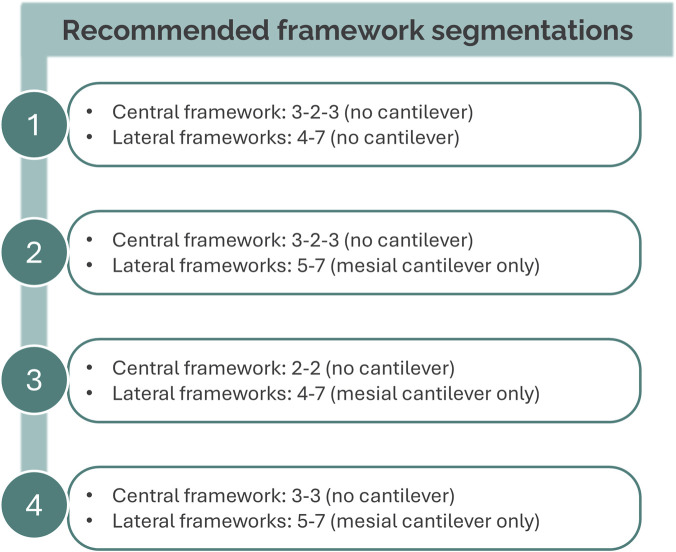
Practical planning guide illustrating the most recommended framework segmentations for full-arch mandibular implant-supported rehabilitation.

Drawing together the foregoing analyses—and acknowledging that individual clinical scenarios will require bespoke solutions—the prioritized layouts in Figure 5 may serve as a practical planning guide. From a mechanical standpoint, a triangulated central span with distal support (3 – 2 – 3 + 4 – 7) is preferable, provided that implants at 3 and 4 are not positioned too closely; adequate inter-implant bone should be preserved to maintain vascularization. Where additional spacing between 3 and 4 is required, a 3 – 2 – 3 + 5 – 7 configuration is favored, with the proviso that any cantilever at 4 is borne by the distal (lateral/straight) span and never by the central span. If triangulation of the central span is not feasible, a 2 – 2 + 4 – 7 arrangement is the next most suitable option, again with the cantilever at 3 supported distally. When necessary, a 3 – 3 + 5 – 7 configuration can be employed under the same caveat regarding the cantilever at 4. These layouts are idealized; anatomical and prosthetic constraints may dictate alternative distributions, but the recommendations provide a pragmatic starting point.

In addition, triangulation becomes particularly advantageous because central implants are often of narrower diameter, either due to anatomical limitations or prosthetic space constraints. Narrower implants exhibit higher stress concentrations under axial and off-axis loading, but a triangulated configuration effectively mitigates these peaks by enhancing load distribution and reducing bending moments on the anterior supports (Anitua et al., 2022; Muangsisied et al., 2021; Valera-Jiménez et al., 2020). Thus, the biomechanical rationale for triangulating the central span is reinforced not only by geometric stability but also by the mechanical behavior of the narrower implants typically employed in this region.

In essence, although segmentation can increase peri-implant bone stresses, careful planning—placing segments at favorable locations and avoiding central-span cantilevers—keeps these increases within controlled limits. From a purely mechanical perspective, the present results therefore help reduce concerns that a segmented framework is intrinsically detrimental in terms of peri-implant stress demand, provided that segmentation is planned appropriately. Importantly, these potential clinical motivations for segmentation are external to the present model and derive from prior clinical/experimental studies, not from our simulations. Accordingly, our results provide a mechanical plausibility check—under the investigated assumptions—complementing previously reported clinical rationale for segmentation.

## 5 Conclusion

Under the investigated implant system and modeling assumptions, segmentation can increase peri-implant bone stresses; however, the present simulations indicate that careful planning—particularly regarding split location, cantilever-bearing span, and central-span support strategy—can keep these increases within controlled limits. Accordingly, the following biomechanically favorable trends were observed under the investigated loading conditions:

- Lateral span loading: When the loaded span lies between splinted supports and no cantilever is present, peri-implant stress levels are similar for complete and segmented frameworks.
- Premolar loading: Stress increases are most pronounced when loading acts on a cantilever. When a cantilever is unavoidable, placing it on a straight lateral span appears mechanically more favorable than placing it on the curved central span.
- Incisor loading (central span): Anterior loading challenges the central span; reducing the effective lever arm by bringing supports closer to the load (e.g., 2–2) or triangulating the central span (e.g., 3–2–3) reduces peri-implant stress compared with non-triangulated central support schemes.

These findings should be interpreted as comparative biomechanical trends from a FEA model, rather than prescriptive clinical rules. Clinical decision-making should also consider anatomical, prosthetic, and biological factors not represented in the model, and future work should evaluate these trends under oblique and cyclic loading and across different implant systems/materials.

## Data Availability

The original contributions presented in the study are included in the article/Supplementary Material, further inquiries can be directed to the corresponding author.
