# Supplementary material for: Three-dimensional finite element analysis of full-arch implant-supported dental restorations: complete vs. segmented frameworks
Source: Front Bioeng Biotechnol. 2026 Apr 30;14:1759007. doi: 10.3389/fbioe.2026.1759007 (PMC13171742; doi:10.3389/fbioe.2026.1759007)
Supplement: Supplementary file 1 [file Table1.docx]

Supplementary Material

Table S 1. *Peak peri-implant bone stress and strain metrics for each implant and configuration.* Values were extracted within the predefined 0.5-mm peri-implant ROI surrounding each implant (see Materials and Methods) and reported as metric-wise extrema within that ROI. Reported outcomes include von Mises equivalent stress ($\boldsymbol{\sigma}_{\boldsymbol{VM}}$, MPa), maximum and minimum principal stresses ($\boldsymbol{\sigma}_{\boldsymbol{1}}$ and $\boldsymbol{\sigma}_{\boldsymbol{3}}$, MPa) and elastic strain measures (equivalent elastic strain, $\boldsymbol{\varepsilon}_{\boldsymbol{eq}}$, mm/mm; principal elastic strains $\boldsymbol{\varepsilon}_{\boldsymbol{1}}$ and $\boldsymbol{\varepsilon}_{\boldsymbol{3}}$, mm/mm). Positive values denote tensile states and negative values denote compressive states. Because different metrics describe different aspects of the multiaxial field, their extrema are not required to occur at the same location within the ROI.

|  | $\boldsymbol{\sigma}_{\boldsymbol{VM}}$(MPa) | $\boldsymbol{\sigma}_{\boldsymbol{1}}$ (MPa) | $\boldsymbol{\sigma}_{\boldsymbol{3}}$ (MPa) | $\boldsymbol{\varepsilon}_{\boldsymbol{eq}}$ (mm/mm) | $\boldsymbol{\varepsilon}_{\boldsymbol{1}}$ (mm/mm) | $\boldsymbol{\varepsilon}_{\boldsymbol{3}}$ (mm/mm) |
| --- | --- | --- | --- | --- | --- | --- |
| Case 1 | | | | | | |
| A implant | 3.3 | 4.9 | -3.0 | 0.000280 | 0.000283 | -0.000283 |
| B implant | 29.4 | 15.8 | -45.3 | 0.002099 | 0.001764 | -0.002509 |
| C implant | 23.3 | 27.6 | -32.8 | 0.001720 | 0.001982 | -0.002135 |
| Case 2 | | | | | | |
| A implant | 2.7 | 0.8 | -3.8 | 0.000219 | 0.000208 | -0.000229 |
| B implant | 29.1 | 15.0 | -44.7 | 0.002093 | 0.001709 | -0.002490 |
| C implant | 24.9 | 28.3 | -33.9 | 0.001848 | 0.001955 | -0.002248 |
| Case 3 | | | | | | |
| A implant | 7.5 | 11.5 | -4.8 | 0.000774 | 0.000641 | -0.000740 |
| B implant | 27.2 | 11.8 | -40.7 | 0.001858 | 0.001617 | -0.002295 |
| C implant | 31.4 | 30.0 | -45.3 | 0.002324 | 0.002185 | -0.002707 |
| Case 4 | | | | | | |
| A implant | 3.1 | 2.0 | -4.1 | 0.000468 | 0.002608 | -0.000503 |
| B implant | 26.1 | 10.9 | -38.8 | 0.001795 | 0.001504 | -0.002205 |
| C implant | 35.0 | 31.0 | -50.2 | 0.002608 | 0.002355 | -0.002897 |
| Case 5 | | | | | | |
| A implant | 23.4 | 9.7 | -36.7 | 0.001721 | 0.001999 | -0.002006 |
| B implant | 27.7 | 13.9 | -40.3 | 0.002020 | 0.001625 | -0.002312 |
| C implant | 22.8 | 36.2 | -6.5 | 0.001042 | 0.001979 | -0.001095 |
| Case 6 | | | | | | |
| A implant | 3.8 | 1.4 | -4.5 | 0.000303 | 0.000346 | -0.000368 |
| B implant | 74.4 | 29.4 | -110.2 | 0.005788 | 0.004147 | -0.006619 |
| C implant | 61.7 | 100.3 | -29.2 | 0.003205 | 0.005427 | -0.002490 |
| Case 7 | | | | | | |
| A implant | 156.0 | 63.9 | -228.6 | 0.013314 | 0.005283 | -0.013316 |
| B implant | 11.1 | 16.8 | -8.6 | 0.000645 | 0.000945 | -0.000665 |
| C implant | 22.2 | 34.5 | -4.0 | 0.001007 | 0.001916 | -0.001130 |
| Case 8 | | | | | | |
| A implant | 13.4 | 6.5 | -19.6 | 0.0011374 | 0.00080195 | -1.41E-03 |
| B implant | 43.1 | 21.3 | -62.9 | 0.0033764 | 0.0030981 | -0.0035742 |
| C implant | 21.1 | 33.2 | -3.9 | 0.00099759 | 0.0018266 | -1.14E-03 |
| Case 9 | | | | | | |
| A implant | 27.6 | 11.9 | -41.2 | 0.001973 | 0.002244 | -0.002333 |
| B implant | 8.2 | 12.2 | -2.5 | 0.000366 | 0.000688 | -0.000567 |
| C implant | 37.5 | 59.6 | -8.0 | 0.001420 | 0.003265 | -0.001392 |
| Case 10 | | | | | | |
| A implant | 97.6 | 115.8 | -139.0 | 0.007627 | 0.006916 | -0.008150 |
| B implant | 17.5 | 26.6 | -7.5 | 0.000418 | 0.001493 | -0.000575 |
| C implant | 26.5 | 41.5 | -4.0 | 0.000879 | 0.002294 | -0.001042 |
| Case 11 | | | | | | |
| A implant | 26.1 | 8.3 | -40.4 | 0.001823 | 0.003319 | -0.002775 |
| B implant | 5.2 | 6.5 | -7.3 | 0.000387 | 0.000454 | -0.000527 |
| C implant | 34.9 | 54.4 | -4.9 | 0.001304 | 0.003021 | -0.001341 |
| Case 12 | | | | | | |
| A implant | 52.9 | 32.2 | -75.5 | 0.004046 | 0.003971 | -0.004420 |
| B implant | 15.1 | 22.0 | -13.2 | 0.0007498 | 0.001260 | -0.000778 |
| C implant | 27.6 | 42.4 | -4.1 | 0.000937 | 0.002371 | -0.001152 |
| Case 13 | | | | | | |
| E implant | 44.6 | 74.2 | -21.6 | 0.001694 | 0.003996 | -0.001986 |
| D implant | 56.6 | 47.7 | -85.3 | 0.004318 | 0.034358 | -0.023258 |
| A implant | 39.4 | 44.2 | -54.7 | 0.003082 | 0.002597 | -0.003241 |
| B implant | 15.9 | 24.2 | -7.5 | 0.000419 | 0.001354 | -0.000521 |
| C implant | 25.2 | 39.5 | -4.1 | 0.000878 | 0.002179 | -0.001090 |
